# Metabolic Bariatric Surgery to Facilitate Access to Renal Transplantation for Patients with Obesity and End- Stage Renal Disease: A National Case Series and Focused Review

**DOI:** 10.3390/jcm15145500

**Published:** 2026-07-14

**Authors:** Sebastian Mitchell, Diana A. Wu, Michael Cheah, Peter Mekhail, Shayanthan Nanthakumaran, Emma MacVicar, Simon Gibson, Ryan Ghita, Brian Joyce, Gillian Drummond, Peter J. Lamb, Rachel A. B. Thomas, Chetan Parmar, Victoria Banwell, Andrew G. Robertson

**Affiliations:** 1 Upper Gastrointestinal and Bariatric Surgery, Royal Infirmary of Edinburgh, Edinburgh EH16 4SA, UK; sebastian.mitchell@nhs.scot (S.M.); michael.cheah@nhs.scot (M.C.); brian.joyce@nhs.scot (B.J.); gillian.drummond@nhs.scot (G.D.); peter.lamb@nhs.scot (P.J.L.); 2 Edinburgh Transplant Unit, Royal Infirmary of Edinburgh, Edinburgh EH16 4SA, UK; diana.wu4@nhs.scot (D.A.W.); rachel.thomas@nhs.scot (R.A.B.T.); victoria.banwell@nhs.scot (V.B.); 3 Upper Gastrointestinal and Bariatric Surgery, Aberdeen Royal Infirmary, Aberdeen AB25 2ZN, UK; peter.mekhail@nhs.scot (P.M.); shayanthan.nanthakumaran@nhs.scot (S.N.);; 4 Upper Gastrointestinal and Bariatric Surgery, Queen Elizabeth University Hospital, Glasgow G51 4TF, UK; simon.gibson@nhs.scot; 5 Glasgow Transplant Unit, Queen Elizabeth University Hospital, Glasgow G51 4TF, UK; ryan.ghita2@nhs.scot; 6 Upper Gastrointestinal and Bariatric Surgery, University Hospital Hairmyres, Glasgow G75 8RG, UK; 7 Upper Gastrointestinal and Bariatric Surgery, Whittington Hospital, London N19 5NF, UK; cparmar@nhs.net

**Keywords:** metabolic bariatric surgery, obesity, end stage renal disease, renal failure, renal transplantation, Scotland

## Abstract

**Background/Objectives**: Obesity is extremely common among patients with end stage renal disease (ESRD) due to shared metabolic risk factors. Renal transplantation is the gold standard treatment for ESRD, however obesity is a major barrier due to increased anaesthetic risks, surgical complications and poorer graft outcomes. The aim of this study was to assess ESRD patients referred for metabolic bariatric surgery (MBS) in Scotland. **Methods**: Data from all adult patients with ESRD who were referred for MBS in Scotland from January 2014 to August 2025 were obtained retrospectively. Categorical data are presented as number and percentage; non-parametric continuous data are presented as median and inter-quartile range. A focused review of comparable published cohorts was performed for context. **Results**: A total of 9 patients with ESRD were referred for MBS over the study period; of these, 5 patients went on to have MBS. Of the patients who underwent MBS, the median age at referral was 47 years, 3 of 5 were female and the median starting weight and body mass index (BMI) were 131 kg and 49 kg/m^2^ respectively. Four patients underwent laparoscopic sleeve gastrectomy, and 1 patient underwent one-anastomosis gastric bypass. The median length of hospital stay was 2 days. The median total percentage weight loss was 33.5% (range 27.7% to 48.1%). Four of the five patients were able to be activated on the transplant waiting list after successfully achieving their target weight after a median of 8.4 months. Of the 4 listed patients, 3 were transplanted with no post-operative complications, and 1 remains active on the waiting list. Across seven comparable cohorts (19 to 198 operated patients), all single- or two-centre, ours had the only national coverage. Listing and transplantation outcomes (4 of 5 listed, 3 of 5 transplanted) were broadly consistent with the range reported in these cohorts, although the small sample precludes formal comparison. **Conclusions**: In this small case series and review, MBS appeared safe and feasible in a highly selected group of patients with obesity and ESRD. MBS facilitates access to renal transplant but is under-utilised in Scotland. We plan to formalise a national treatment pathway to streamline access to bariatric services for patients with ESRD and enable more patients to undergo renal transplantation.

## 1. Introduction

Obesity is an important risk factor for both the development and progression of chronic kidney disease (CKD), ultimately leading to end stage renal disease (ESRD) and the need for dialysis or renal transplantation (RT). Both obesity and CKD commonly co-exist with other metabolic diseases including type 2 diabetes mellitus, cardiovascular disease and hypertension, all of which increase the risk of early mortality.

In the UK, obesity is extremely common among patients with ESRD, with an estimated prevalence of around 33% [1]. For most patients with ESRD, RT is the gold standard treatment, leading to improved survival and quality of life compared with dialysis [2,3]. Obesity is a major barrier to transplantation due to the increased anaesthetic risks [4], surgical complications and higher risk of graft failure [5]. This sits alongside the well-described ‘obesity paradox’ in dialysis, whereby a higher BMI is associated with better survival on dialysis [6]. This reverse epidemiology is, however, specific to the dialysis state and does not extend to transplantation: higher pre-transplant BMI remains associated with worse peri-operative and graft outcomes [5]. The paradox therefore does not weaken the case for weight reduction to enable transplant access: the durable survival advantage lies with transplantation, which obesity obstructs. Many centres exclude patients from transplantation on the basis of body mass index (BMI) thresholds, but thresholds vary considerably between different centres, leading to inequity in access to transplantation [7].

Currently, there is no clear treatment pathway in the UK for patients who fall outside the criteria for RT due to their weight, but who would otherwise have been considered suitable for a kidney transplant. Patients are frequently asked to lose weight to a target level before they can undergo RT, but limited support is available. Losing weight in the context of ESRD is challenging, due to the dietary restrictions required in renal failure, fluid shifts and fatigue, limiting the ability to exercise. Non-surgical options exist but are limited in this population. Medical and lifestyle (tier 2/3) weight-management programmes achieve only modest results, and GLP-1 receptor agonists, now licensed across CKD stages, including dialysis, achieve substantially smaller weight loss in this population than MBS [8]. Against this backdrop, metabolic bariatric surgery (MBS) offers the most reliable and durable route to the weight loss required for transplant eligibility [9]. Metabolic bariatric surgery (MBS) has been shown to be a safe and efficacious option for patients with obesity and ESRD and can facilitate access to RT [9]. Despite this, a recent survey revealed that only 1 out of 23 RT centres in the UK regularly refers patients for MBS [7].

In Scotland, with a population of 5.5 million, around 565 patients start dialysis and around 240 patients receive a kidney transplant each year [10]. In general, patients must complete a medical weight management (Tier 3) programme overseen by a specialist multi-disciplinary team (MDT) prior to being considered for MBS. The aim of this study was to assess ESRD patients referred for MBS in Scotland and describe their outcomes, in order to inform a national initiative to formalise a treatment pathway for patients who are deemed unsuitable for RT due to their weight.

## 2. Materials and Methods

### 2.1. Data Collection

Patients were referred for MBS if they had a BMI greater than 35 kg/m^2^ with obesity-related comorbidities, or a BMI greater than 40 kg/m^2^. For activation on the transplant waiting list, a target BMI threshold of 35 kg/m^2^ was generally applied, with clinical-assessment discretion exercised by the MDT in keeping with the individualised approach described above. Data capture covered all 6 Scottish MBS centres; of these, 3 centres performed MBS in eligible patients during the study period and 3 did not.

Data from all adult patients with ESRD who were referred for MBS in Scotland from January 2014 to August 2025 were obtained from electronic databases and medical notes. Ethical approval was gained for the collection of patient data. Collected data included age; sex; primary renal disease; hypertension; type 2 diabetes mellitus; need for dialysis; weight and BMI at time of referral to weight management services, at time of surgery, at time of listing for renal transplantation and at time of renal transplantation, type of metabolic bariatric surgery; length of hospital stay; complications of metabolic bariatric surgery; complications of renal transplant surgery; percentage total weight loss; time to listing for renal transplant; time to renal transplant; current renal transplant status; creatinine and eGFR at last follow-up; and reasons for not undergoing metabolic bariatric surgery. Post-operative complications were classified using the Clavien–Dindo classification.

Categorical data are presented as number (and percentage where appropriate), non-parametric continuous data are presented as median and inter-quartile range. Follow up was defined from the date of first referral to weight management services to last documented clinical interaction. The data censor date was August 2025. As Scotland has a geographically defined renal population managed within a small number of services, complete follow-up was achievable and there were no losses to follow-up.

### 2.2. Surgical Technique

Laparoscopic sleeve gastrectomy (LSG) was the procedure of choice, in keeping with the European Renal Association guidelines, to reduce the risk of allograft oxalosis or issues with absorption of immunosuppressive medications [11]. LSG was performed in a standard fashion, which has been described previously: the stomach was mobilized 5 cm from the pylorus to the left crus. A 34 Fr gastric calibration tube was inserted and the stomach was stapled using a purple 60 mm Endo-GIA (Medtronic, Minneapolis, Minnesota, USA) and Tan 60 Endo-GIA (Medtronic, Minneapolis, Minnesota, USA). A 2/0 PDS suture (Johnson & Johnson, Raritan, New Jersey, USA) was used to buttress the lower staple line to reduce the chance of post-operative haemorrhage [12].

Laparoscopic one-anastomosis gastric bypass (OAGB) was performed in a standard fashion. This patient had undergone an LSG elsewhere approximately 10 years previously and was converted to an OAGB to achieve further weight loss. OAGB was selected in this single case specifically because the patient had already undergone LSG and required a further weight-loss procedure; a malabsorptive option was therefore unavoidable. The attendant risks of altered enteric oxalate handling and potential effects on immunosuppressive drug absorption were recognised and, following transplantation, would be mitigated through close therapeutic drug monitoring and renal surveillance. The gastric pouch was created from the sleeve in the horizontal portion of the lesser curvature, to ensure the longest possible gastric pouch, a portion of the antrum is incorporated in the gastric pouch. This was created using a 45 mm Endo-GIA. The biliopancreatic limb was measured to 150 cm and an antecolic, antegastric gastrojejunostomy was formed using a 45 mm Endo-GIA and the enterotomy was sutured. Petersen’s defect was subsequently closed with an Endohernia Stapler.

### 2.3. Focused Review of Comparable Studies

#### 2.3.1. Search Strategy

PubMed (National Library of Medicine, Bethesda, MD, USA) was searched from inception to 16 May 2026 (the clinical data censor date for the case series was August 2025) using terms relating to metabolic bariatric surgery, kidney transplantation or transplant candidacy, and obesity or chronic kidney disease. The full search strategy was: (“Bariatric Surgery”[Mesh] OR “bariatric surgery”[tiab] OR “weight loss surgery”[tiab] OR “gastric bypass”[tiab] OR “sleeve gastrectomy”[tiab] OR “gastric banding”[tiab] OR “metabolic surgery”[tiab]) AND (“Kidney Transplantation”[Mesh] OR “kidney transplant*”[tiab] OR “renal transplant*”[tiab] OR “transplant waitlist”[tiab] OR “transplant wait list”[tiab] OR “waiting list”[tiab] OR “transplant candidacy”[tiab] OR “transplant eligibility”[tiab] OR “transplant listing”[tiab] OR “listed for transplant*”[tiab]) AND (“Obesity”[Mesh] OR obes*[tiab] OR “body mass index”[tiab] OR BMI[tiab] OR “Renal Insufficiency, Chronic”[Mesh] OR “chronic kidney disease”[tiab] OR “end stage renal”[tiab] OR ESRD[tiab] OR dialysis[tiab] OR CKD[tiab]).

#### 2.3.2. Eligibility Criteria

Studies were eligible for inclusion if they reported patients referred for, assessed for, or undergoing MBS as a strategy to improve access to kidney transplantation. Eligible populations included patients with advanced chronic kidney disease, end-stage kidney disease, or dialysis dependence who were not yet listed or activated for kidney transplantation because obesity represented a barrier to transplant eligibility or waitlisting. Studies were required to follow patients beyond MBS referral or surgery and to report at least one transplant-access outcome, such as achievement of transplant eligibility, waitlisting, activation on the transplant waiting list, or subsequent kidney transplantation. Studies were excluded if they included 15 or fewer patients, reported patients who were already listed or transplanted before MBS without a clear transplant-access indication, did not report MBS in the context of kidney transplant candidacy, or lacked sufficient clinical outcome data. Case reports, conference abstracts without extractable outcomes, narrative reviews, editorials, and commentaries were excluded. Where multiple studies appeared to use overlapping or duplicate source populations, the study with the larger sample size or more complete outcome reporting was retained.

#### 2.3.3. Study Selection

Titles, abstracts and full texts were screened independently by two reviewers against the eligibility criteria, with disagreements resolved by consensus, and reasons for exclusion at full-text stage were recorded.

#### 2.3.4. Data Extraction

Data were extracted on study characteristics and outcomes relevant to comparison with the Scottish cohort. Extracted variables included country, study design, sample size, patient population, baseline BMI, renal disease status, dialysis status, transplant listing or eligibility status, MBS procedure performed, follow-up duration, weight-loss outcomes, transplant listing outcomes, kidney transplantation outcomes, perioperative complications, and mortality where reported.

#### 2.3.5. Narrative Comparison with the Scottish Cohort

Findings from the focused review were summarised narratively and compared with the Scottish cohort to identify similarities and differences in patient characteristics, referral pathways, procedural outcomes, weight-loss response, access to transplant listing, kidney transplantation outcomes, complications, and mortality. No formal meta-analysis was performed, as the review was intended to provide contextual comparison and the available studies varied in design, population, procedure type, and reported outcomes.

## 3. Results

A total of 9 patients with ESRD were referred for MBS at 3 units in Scotland over the study period; of these, 5 patients went on to have MBS. The baseline characteristics of all patients are shown in Table 1. Of the 5 patients who underwent MBS, the median age at referral was 47 years, 3 of 5 were female and the median starting weight and body mass index (BMI) were 131 kg and 49 kg/m^2^ respectively.

### 3.1. Patients Who Underwent Metabolic Bariatric Surgery

Five patients underwent MBS (Table 2). The starting BMI ranged from 43.3 to 59.8 kg/m^2^. Four patients underwent LSG and 1 patient who had undergone LSG approximately 10 years previously underwent conversion to OAGB. The median length of hospital stay was 2 days. Only 1 patient suffered a post-operative complication of a chest infection.

Four of five patients were activated on the transplant waiting list after successfully achieving their target weight after a median of 8.4 months. One patient has not yet been activated on the waiting list but is on track to achieve their target weight. Of the 4 listed patients, 3 have been transplanted after a median of 5.4 months after listing (median 13.1 months after MBS) and 1 remains active on the waiting list. All three transplants were elective, planned procedures; one received a living-donor kidney and two a deceased-donor kidney. There were no post-operative complications. Of the 3 transplanted patients, 2 have good transplant function and 1 has temporary impairment in renal function in the post-partum period due to a complicated delivery.

**Figure 1:**
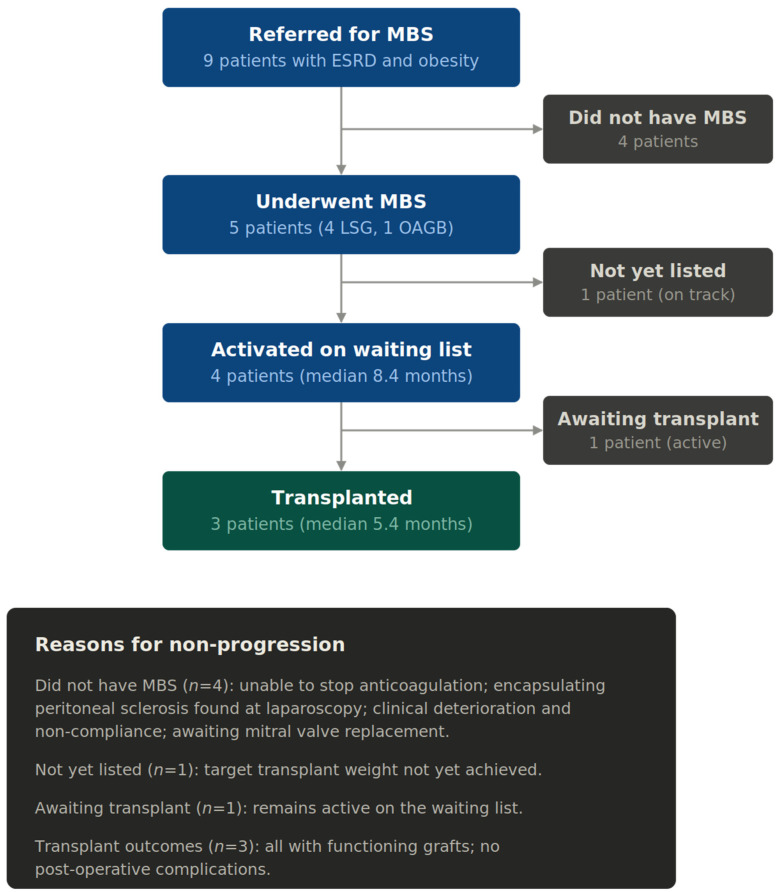
Flow of patients with ESRD and obesity through the metabolic bariatric surgery and renal transplantation pathway.

The median total percentage weight loss (TWL) was 33.5% (range 27.7% to 48.1%). Per-timepoint weight, BMI and renal function data for each patient are provided in Supplementary Table S1. Two of four patients with pre-operative HTN achieved remission post MBS. Neither of the two patients with pre-operative T2DM achieved remission post MBS. There were no re-operations or mortalities in the MBS patient group. The total length of follow up from first referral to weight management services to last follow up ranged from 1.8 to 11.5 years, with a median of 4.75 years. The flow of patients through referral, surgery, listing and transplantation is shown in Figure 1.

### 3.2. Patients Who Did Not Undergo Metabolic Bariatric Surgery

Four patients did not undergo MBS (Table 3). The starting BMI ranged from 42.3 to 53.8 kg/m^2^. Patient 6 was unable to undergo MBS as they were unable to stop warfarin due to recurrent thrombosis in the arterio-venous graft required for dialysis. Due to the recurrent difficulties with vascular access, there was concern that the patient would be left in a situation where dialysis was no longer possible. Therefore, the transplant MDT decided to list the patient at a BMI of 38 kg/m^2^ and accept the higher risks associated with obesity. The patient underwent a transplant that was complicated by a perinephric haematoma, but recovered well. The patient was started on a GLP-1 receptor agonist post transplantation which has resulted in 42 kg (35%) weight loss in 1.5 years and a 20% increase in renal function. Patient 7 had a functioning renal allograft from a transplant 12 years previously, with gradual decline in graft function, and was referred for MBS to facilitate weight loss ahead of re-listing for re-transplantation. They got as far as the operating room for a planned LSG; however, at laparoscopy the discovery of encapsulating peritoneal sclerosis resulted in abandonment of the procedure. Patient 8 started the weight management programme but became unsuitable for both MBS as well as transplant surgery due to a deterioration in clinical condition as a result of vascular access issues and non-compliance with treatment. Patient 9 started the weight management programme but prior to being assessed for MBS was found to require a mitral valve replacement. Patient 9 has lost a good amount of weight through lifestyle interventions and will be reassessed for RT once recovered from cardiac surgery. Of the four patients who did not undergo MBS, two received or were referred for GLP-1 receptor agonist therapy (Patients 6 and 7). Patient 8 was not commenced on a GLP-1 receptor agonist owing to progressive clinical deterioration and treatment non-compliance, and Patient 9 also was not, as the immediate clinical priority was cardiac surgery; GLP-1 receptor agonist therapy may be considered following recovery. These patients were clinically followed up by their renal teams for a range of 2–7 years.

### 3.3. Summary of Comparable Studies

The focused review identified seven published cohorts reporting MBS as a strategy to facilitate kidney transplantation in patients with obesity and advanced kidney disease, against which the present series is compared in Table 4 and Table 5 and the Supplementary Files for a flowchart of selected studies [13,14,15,16,17,18,19]. Cohort sizes ranged from 19 to 198 operated patients, and all were single- or two-centre studies; the present series is the only one with national coverage. LSG was the predominant procedure in most cohorts, although two series used RYGB as the principal operation. Access to the transplant waiting list ranged from approximately 36% to 81% of operated patients across the published cohorts, and the proportion subsequently transplanted ranged from 21% to 67%. In the present series, 4 of 5 operated patients were listed and 3 of 5 transplanted; these counts fall within the range reported elsewhere, but the small sample size precludes any meaningful comparison of efficacy. Reported peri-operative complication rates also varied widely (from around 1% to 26%).

## 4. Discussion

This study demonstrates that MBS is under-utilised in the ESRD population in Scotland, with only 9 patients referred and only 5 patients undergoing MBS in the last decade. Despite these small numbers, our experience suggests MBS was safe and feasible in selected ESRD patients and facilitated access to RT.

Obesity is very common in the ESRD population and poses additional challenges in the optimal treatment of these patients. RT is the treatment of choice for ESRD due to significantly improved survival, quality of life and cost effectiveness, compared with dialysis. However, patients with obesity are often deemed unsuitable for transplantation due to the higher anaesthetic risks [4], surgical complications and poorer graft outcomes [5]. UK Renal Association guidelines suggest that while a BMI of more than 30 kg/m^2^ is not an absolute contra-indication to transplantation, each case should be screened rigorously and considered individually, and that individuals with a BMI of over 40 kg/m^2^ are less likely to benefit from transplantation [20].

In Scotland, weight management services follow a four-tiered approach recommended by UK national guidelines, with wide variation in provision across health boards in Scotland and the wider UK [21,22,23]. It is therefore unsurprising that no national strategy exists for managing obesity in patients with ESRD. In a recent survey of all 23 UK RT centres, only 9 had access to MBS and most had referred no patients in the previous year; our finding of only 9 ESRD referrals in a decade is in keeping with this [7]. In a meta-analysis of 3.6 million patients, MBS had a peri-operative mortality of 0.08% in the general population, falling to 0.05% for LSG, the procedure of choice in renal transplant candidates [24]. Historically, there may have been a perception that patients with ESRD carry prohibitively high peri-operative risks for MBS. However, contemporary studies show that the risks are acceptable and that patients have good outcomes [9,13,25]. Despite this, a reluctance among bariatric surgeons to operate on patients with ESRD persists in practice, reflecting the perceived peri-operative risk and the concern that intervention may jeopardise vascular access. This surgeon-side hesitancy, alongside the service-level variability described above, is a recognised contributor to under-referral and under-utilisation, and is precisely the kind of barrier a structured national pathway, with agreed selection criteria and shared MDT governance, is designed to overcome [7]. Under-utilisation is not solely a supply-side problem. In comparable cohorts, a substantial proportion of referred patients did not proceed to surgery; for example, only around half of those referred were operated in two series reporting a clean referral denominator, reflecting a mix of patient declining or disengagement and clinical contraindication [14,15]. Demand- and engagement-side factors therefore also contribute to the low operative numbers. In contrast to previous UK series, which have reported single-centre experiences with relatively short follow-up, our study provides national coverage across all Scottish transplant and bariatric centres and longer-term follow-up (median 4.75 years, up to 11.5 years), albeit in a smaller and uncontrolled cohort [15].

Our findings are broadly concordant with the published literature. Across comparable cohorts, MBS consistently enabled a substantial proportion of patients with obesity and advanced kidney disease to access the transplant waiting list and proceed to transplantation, with weight-loss outcomes in keeping with those reported elsewhere. Direct comparison is limited by marked methodological heterogeneity: studies differ in their inclusion criteria, the BMI anchor used, the weight-loss metric reported (total weight loss versus excess weight loss), the predominant procedure, and the follow-up window, and few report a clean referral-to-operation denominator. The comparator cohorts also point to a group that is easily overlooked: patients operated as a bridge who never reach transplantation, lost between surgical and transplant follow-up, with delisting and pre-transplant deaths reported in the larger series [13,17]. In our cohort the non-progression lay upstream instead: four of five operated patients were listed and the fifth is near target, whereas those who stalled were largely those never operated on. The wide range of reported peri-operative complication rates likely reflects, at least in part, differences in complication definitions and follow-up duration between studies; however, in the absence of standardised, comparative data, genuine differences in safety between cohorts and procedures cannot be excluded. Our series is distinguished from prior work less by the magnitude of its outcomes than by its national scope and its longer follow-up, which exceeds that of most comparable cohorts; against larger single-centre series such as Kassam et al. and Hajjar et al. however, our cohort is considerably smaller and uncontrolled [13,17]. The listing and transplantation outcomes in our series should therefore be interpreted in the context of a small, highly selected national cohort rather than as evidence of superior efficacy.

There is an absence of direct comparative data or randomised trials comparing the different metabolic bariatric surgical procedures, i.e., laparoscopic sleeve gastrectomy, laparoscopic Roux-en-Y gastric bypass and laparoscopic one-anastomosis gastric bypass. However, there exists several valid and important reasons to favour laparoscopic sleeve gastrectomy over other operative metabolic bariatric procedures in end stage renal disease patients. Besides published evidence showing a benefit for renal transplant graft survival, compared with Roux-en-Y gastric bypass, sleeve gastrectomy has not been shown to impair immunosuppressive drug absorption and does not affect oxalate absorption as intestinal absorption has not been modified. Although laparoscopic sleeve gastrectomy may be inferior in terms of percentage total weight loss and remission of type 2 diabetes mellitus, the benefits in terms of preserving immunosuppressive drug absorption and a reduced risk of transplant oxalosis make it our favoured and recommended procedure [11]. The single patient in our series who underwent OAGB had previously had an LSG and required a further weight-loss procedure, for which a malabsorptive operation was unavoidable; this case illustrates that, while LSG remains our default, procedure selection must occasionally accommodate prior surgical history. In such patients, the recognised risks of altered enteric oxalate handling and impaired immunosuppressive absorption warrant heightened post-transplant surveillance: malabsorption and altered gastrointestinal transit after OAGB may affect tacrolimus exposure, compound mycophenolate-associated gastrointestinal toxicity, and influence valganciclovir bioavailability, mitigated through close therapeutic drug monitoring and individualised dosing [11]. Notably, this patient has not yet been transplanted, so these interactions remain anticipatory rather than observed in our series.

Which ESRD patient with obesity should be offered MBS, and when, is not resolved by a BMI threshold alone. BMI is an imperfect surrogate of transplant risk: although a higher BMI carries greater peri-operative complexity and worse graft outcomes, the competing risks of remaining on dialysis are dynamic and vary widely between individuals [5,20]. In our practice, selection rests on the patient’s overall clinical context rather than a single number: dialysis adequacy and tolerance, vascular-access integrity, comorbidity and physiological fitness, body habitus, prior weight-loss attempts, anticipated waiting time, living-donor availability, expected donor characteristics, and the centre’s experience in transplanting patients with obesity. Together, these determine whether the operative risk of transplantation is outweighed by the cumulative risk of continued dialysis. Because all transplants in our series were elective and planned, there was a defined window in which to optimise weight before listing. One operated patient was not dialysis-dependent; in such pre-dialysis candidates the risk–benefit balance differs, since the vascular-access and dialysis-tolerance constraints do not yet apply and MBS may instead secure pre-emptive transplantation. Without intervention, many such patients remain ineligible despite obesity being their principal modifiable barrier; conversely, transplantation may be justified when the risks of ongoing dialysis outweigh the incremental operative risk of an elevated BMI. Obesity is therefore best regarded as one component of a comprehensive risk assessment, not an absolute contraindication [11,20]. Patient 6 illustrates this: listed and transplanted at a BMI of 38 without prior MBS, because the threat of losing dialysis access outweighed the obesity-related operative risk. MBS is thus one option within the pathway rather than a universal prerequisite, and the dialysis-access calculus can justify proceeding directly to transplantation in selected patients.

In our series there was only one minor post-operative complication (chest infection) after MBS and all 5 patients are alive after a median follow up of 4.75 years from the time of first referral to the weight management programme. All patients accomplished excellent total weight loss (median 46 kg, 33.5%) to a level much greater than what is typically achieved through non-surgical interventions, although given the small number of patients these summary figures should be interpreted with caution and not extrapolated as broadly generalisable. For example, in Scotland, the mean weight loss through tier 2 services is 2% and through tier 3 services is 5% [26]. Although there is increasing interest in the use of GLP-1 receptor agonists, there is limited evidence on their use in the ESRD population and their long term outcomes. One study reported an average of 4.6% total weight loss with GLP-1 receptor agonist use in advanced CKD patients [8] and another reported that, among dialysis patients with obesity and type 2 diabetes, bariatric surgery was associated with a substantially increased likelihood of transplant waitlisting and with long-term survival benefit, and that eligible candidates were more likely to undergo bariatric surgery than to commence a GLP-1 receptor agonist to qualify for transplantation [27]. That analysis was a US registry-based claims cohort study; our study complements it by providing real-world national clinical data, including the practical barriers to MBS and the degree of under-utilisation in current practice, which a population-level claims analysis cannot capture.

In our study, MBS facilitated access to the RT waiting list in 4 out of 5 patients, with the fifth patient on track to achieve their target weight for listing. This was achieved within a very short median time frame of 8.4 months. With non-surgical weight loss interventions, patients may spend a long time trying to achieve a target weight for listing, during which time they may accumulate extra morbidity and become unfit for transplantation, thus missing their window of opportunity.

Three patients have been transplanted, all with functioning grafts. These are young patients (median age 47) who would otherwise face a life of dialysis and its associated morbidity. The 5-year survival of patients with obesity on dialysis is around 40% [6], and some may not have survived without transplantation, although our study was not designed to assess survival. MBS also confers benefits including remission of type 2 diabetes and hypertension [12]. In our series, two of four patients with pre-operative hypertension achieved remission, but neither of the two with type 2 diabetes did, likely reflecting the diabetogenic effects of immunosuppression, including high-dose glucocorticoids and calcineurin inhibitors.

There are substantial cost savings associated with MBS and transplantation compared with dialysis. It is estimated that 5 years of hospital haemodialysis costs around £170,000 per patient in the UK, while the cost of renal transplantation with 5 years of follow-up care is £52,000 and the cost of MBS is £9000 [28,29]. This equates to potential cost savings to the National Health Serviceof £109,000 per patient. This is likely an underestimate as it does not include the cost of additional morbidity acquired whilst on dialysis nor the costs of unemployment for patients, their families and the wider economy. These figures are derived from published cost estimates and are illustrative; our study did not perform a formal cost analysis.

Several factors explain why patients may not progress to MBS in the context of ESRD. Difficult vascular access is common in haemodialysis patients, and any intervention may threaten a patient’s only access and leave them unable to dialyse. Encapsulating peritoneal sclerosis, a rare complication of long-term peritoneal dialysis causing dense adhesions and small-bowel encapsulation, can also preclude surgery, as can the less severe intra-abdominal adhesions frequently seen after prolonged peritoneal dialysis [30].

The main limitations of this study are its small sample size and retrospective design, and it is best regarded as a descriptive case series rather than a comparative study. Importantly, the cohort was highly selected, and our findings are subject to referral bias, inter-centre variation in practice, MDT-based selection of surgical candidates, and survivorship bias; only patients judged fit enough to proceed underwent MBS, and the favourable outcomes observed likely reflect this selection rather than demonstrating that MBS is broadly safe across the wider ESRD population with obesity. There was a no comparator group, and because outcomes are based on very small numbers, proportions are unstable; we have therefore presented raw counts throughout, and these figures should not be extrapolated. The accompanying review was a focused, non-systematic comparison undertaken for context: it did not involve a formal risk-of-bias assessment or certainty (e.g., GRADE) grading, and the comparator studies were methodologically heterogeneous in design, population, procedure mix, BMI anchor, and follow-up. We did not assess body composition or nutritional status; loss of lean mass or sarcopenia accompanying rapid weight loss is an unmeasured consideration, and our numbers are too small to detect any rejection or infection signal. These factors substantially limit the generalisability of our findings. The small number of transplants also precludes any analysis of how donor type or transplant timing influenced outcomes. However, the strengths of the study are the long follow up time and that it highlights this problem in our country, raising awareness of the benefit of MBS in this patient population. Larger studies, ideally using national registry or database linkage, are required to fully assess the short- and long-term outcomes of MBS in patients with ESRD.

## 5. Conclusions

In this small, retrospective case series, MBS appeared safe and feasible in a highly selected group of patients with ESRD and obesity and facilitated access to RT. While these descriptive findings are encouraging, they should be interpreted cautiously given the small cohort and selection bias, and cannot be generalised to the wider ESRD population. MBS nonetheless appears to be under-utilised in Scotland. It could also lead to substantial cost savings for the health service. We plan to use this data to formalise a national treatment pathway that will streamline access to bariatric services for patients with ESRD, including structured follow-up of those who do not progress to transplantation, and ultimately enable more patients to undergo RT.

## Figures and Tables

**Table 1:** Baseline characteristics.

Patient Characteristics at the Time of Referral for MBS	All Patients (*n* = 9)	MBS Group (*n* = 5)
Age, median (IQR)	47 (42–53)	47 (47–53)
Female, *n* (%)	6	3
Primary renal disease, *n* (%)		
-Polycystic kidney disease	2	1
-Type 2 diabetes mellitus	2	1
-Glomerulonephritis	1	1
-Calculus nephropathy	1	1
-Reflux nephropathy	1	0
-Unknown	2	1
Hypertension, *n* (%)	8	4
Type 2 diabetes, *n* (%)	3	2
Dialysis, *n* (%)	6	4
Starting weight, kg, median (IQR)	133 (129–161)	131 (129–137)
Starting BMI, kg/m^2^, median (IQR)	49 (43–54)	49 (44–51)

MBS: metabolic bariatric surgery, IQR: inter-quartile range, BMI: body mass index.

**Table 2:** Metabolic bariatric surgery (MBS) group outcomes.

	Patient 1	Patient 2	Patient 3	Patient 4	Patient 5
Starting weight (kg)	131	137	204.5	129.4	117.6
Type of MBS	LSG	OAGB	LSG	LSG	LSG
Length of hospital stay (days)	2	2	2	3	5
Complications of MBS	Nil	Nil	Nil	Nil	Chest infection
Activated on transplant waiting list	Yes	Yes	No ^1^	Yes	Yes
Transplanted	Yes	No	No	Yes	Yes
Complications of transplant	Nil	-	-	Nil	Nil
Current transplant status	Good function	-	-	Good function	Temporary impairment ^2^
Total weight loss (kg) ^3^	63	46	68.5	42.2	32.6
Total weight loss (%) ^3^	48.1	33.6	33.5	32.6	27.7
Time, referral to MBS (years)	2.7	1.9	1.1	0.5	1.2
Time, MBS to listing (months)	9.2	7.6	-	5.9	9.8
Time, listing to transplant (months)	5.4	-	-	7.2	2.5
Length of follow up (years) ^4^	8.8	2.7	1.8	11.5	4.75

^1^ Transplant weight not yet achieved (136 kg/BMI 40 kg/m^2^ at last review). ^2^ Temporary impairment in renal function in the post-partum period due to a complicated delivery. ^3^ For patients not transplanted, total weight loss is calculated from the most recent recorded weight (weight at listing for Patient 2; weight at last review for Patient 3). ^4^ Time from first referral to weight management service to last follow up. MBS: metabolic bariatric surgery, LSG: laparoscopic sleeve gastrectomy, OAGB: one anastomosis gastric bypass.

**Table 3:** Non-metabolic bariatric surgery group outcomes.

	Patient 6	Patient 7	Patient 8	Patient 9
**Starting weight (kg)**	133	161	162.3	100.2
**Starting BMI (** **kg/m^2^)**	42.5	53.8	53.7	42.3
**Reason for not undergoing MBS**	Unable to stop warfarin as required to maintain patent AVG for dialysis	Encapsulating peritoneal sclerosis seen during attempt at LSG	Issues with vascular access and non compliance, deteriorated and became unsuitable for surgery	Required MVR for rheumatic heart disease
**Outcome**	Transplanted at BMI 38, complicated by perinephric haematoma, now has good function	Previous transplant 12 years earlier with declining graft function; referred for MBS to facilitate re-transplantation. Procedure abandoned. Graft function currently moderate	Died	Lost weight without MBS, to be reassessed for transplant after they recover from MVR
**GLP-1 receptor agonist use**	Yes post-transplant, resulting in 20% improvement in renal function	Has been referred	No	No

BMI: Body Mass Index, MBS: Metabolic Bariatric Surgery, AVG: arterio-venous graft, LSG: Laparoscopic sleeve gastrectomy, MVR: mitral valve replacement, GLP-1: glucagon-like peptide-1.

**Table 4:** Comparison of the present series with previously published cohorts of metabolic bariatric surgery in patients with obesity and end-stage renal disease-baseline characteristics, procedure and weight loss.

Study	Setting & Period	Patients, *n* (Referred → Operated)	Procedure(s)	Age at MBS Referral, Years	Female, % (Operated Cohort)	BMI at MBS Referral, kg/m^2^	Weight Loss
Current study	Scotland, UK; national, 3 centres; retrospective; 2014–2025	9 → 5	LSG (4); OAGB (1)	47	60 (3/5)	49	33.5% TWL from referral weight (range 27.7–48.1%)
Kassam et al., 2020 [13]	USA; 2 centres (Cincinnati); prospectively collected, retrospective review; 2011–2018	198 operated (ESRD; referred NR)	SG (198)	54.1 ± 11.1 (mean; whole cohort incl. CKD)	58 (whole cohort, incl. CKD)	44.0 ± 6.3 (mean; at surgery, whole cohort)	18.9% TWL (mean); 38.2% EWL; BMI 44.0 → 36.7
Kukla et al., 2024 [14]	USA; single centre (Mayo, TRANSMET); retrospective; 2020–2023	106 → 54	SG (54)	51.5 ± 10.3 (mean)	44.4 (24/54)	41.7 ± 3.6 (mean)	21.3% TWL at 12 months (mean); BMI 41.7 → 32.6
Bosch et al., 2024 [15]	England, UK; single centre (Whittington, London); retrospective; 2013–2021	31 → 19	SG (18); RYGB (1)	52 (median)	68 (13/19)	46.2 ± 4.9 (mean)	71.2% EBWL at 2 years (mean)
Soliman et al., 2021 [16]	USA; single centre (Houston Methodist, MBSAQIP); retrospective; 2011–2018	38 operated (referred NR)	LRYGB (24); LSG (14)	49 (median; at surgery)	52.6 (20/38)	44.2 ± 6 (mean; at surgery)	23.3% TWL at 12 months (mean); BMI ≤35 in 75.8%
Hajjar et al., 2021 [17]	Canada; 2 centres (Montréal); retrospective; 2013–2020	80 operated (referred NR)	LSG (80)	50 (median; at LSG)	41.3 (33/80)	43.7 (median; at LSG)	EBWL 55.5% at 1 year (median); BMI 43.7 → 33.7
Yemini et al., 2019 [18]	Israel; single centre (Beilinson); retrospective; 2009–2017	24 operated (referred NR)	LSG (17); LRYGB (7)	56 (median; at surgery)	33.3 (8/24)	41.5 ± 0.8 (mean; at surgery)	29% TWL (mean); 66% EWL; BMI 41.5 → 29
Thomas et al., 2018 [19]	USA; single centre (Miami); retrospective; 2009–2014	31 analysed (33 operated; referred NR)	LRYGB (31)	47 (median; at surgery)	54.8 (17/31)	43.2 (median; at surgery)	36% TWL (mean); 72.8% EWL; BMI 43.5 → 28.1

Abbreviations: BMI, body mass index; CKD, chronic kidney disease; EBWL, excess body weight loss; ESRD, end-stage renal disease; EWL, excess weight loss; LRYGB, laparoscopic Roux-en-Y gastric bypass; LSG, laparoscopic sleeve gastrectomy; MBS, metabolic and bariatric surgery; MBSAQIP, Metabolic and Bariatric Surgery Accreditation and Quality Improvement Program; NR, not reported; OAGB, one anastomosis gastric bypass; RYGB, Roux-en-Y gastric bypass; SG, sleeve gastrectomy; TWL, total weight loss.

**Table 5:** Comparison of the present series with previously published cohorts of metabolic bariatric surgery in patients with obesity and end-stage renal disease-transplant pathway, safety and follow-up.

Study	Access to Transplant Waiting List	Transplanted	Reasons for Attrition	MBS Peri-Operative Complications	Post-Transplant Surgical Complications	Mortality	Length of Follow-Up from Referral
Current study	4/5; median 8.4 months after MBS	3/5; median 13.1 months after MBS	Not operated (4/9): vascular access (2), peritoneal sclerosis (1), cardiac surgery (1). Not transplanted (2/5): not yet listed (1), awaiting transplant (1)	1/5 (chest infection); no re-operation	None (0/3 transplanted)	None (0/5) over 4.75-year median follow-up	4.75 years (range 1.8–11.5)
Kassam et al., 2020 [13]	71/198 (35.9%) waitlisted	45/198 (23%); 15 LD/30 DD	Not operated (256/499): breakdown NR. Of 71 listed: 16 removed-weight regain (6), deterioration (3), lost to follow-up (3), requested removal (3), death (1). 127 ESRD not yet listed	2/243 (1.2%): dehydration (1), pulmonary embolism (1); no 90-day mortality	NR	0 at 90 days; 16/198 (8.1%) over full follow-up; mean 2.3 y to death	2.3 ± 1.5 years (mean)
Kukla et al., 2024 [14]	37/54 (69%) waitlisted	20/54 (37%); median 20.9 months after SG	Non-operated (52): declined SG, or psychiatric/medical contraindication, or no insurance coverage (breakdown NR)	3/54 (5.6%): bleeding requiring transfusion (2), GI perforation/anastomotic leak (1)	NR	NR	15.5 months (IQR 6.4–23.9)
Bosch et al., 2024 [15]	11/19 (58%) reached target (5 waitlisted + 6 transplanted)	6/19 (32%)	Not operated (12): BS refused (5), lost weight without BS (2), unfit (2), not documented (3). Not listed post-BS (8): eGFR above threshold (4), cardiac comorbidity (2), death (2)	1/19 early minor (wound infection); no major <30-day. 3 late major (>1 yr): sleeve migration (2), internal hernia (1)	NR	0% at 30 days; 2/19 (11%) long-term (both unrelated to BS)	NR (outcomes reported to 2 years)
Soliman et al., 2021 [16]	18/38 (47%) listed	8/38 (21%)	Referral-stage attrition NR. Not listed/transplanted attributed to uncontrolled comorbidity, socio-economic/insurance factors, psychosocial issues, waitlist times	No intra-operative complications; 30-day readmission 2/38 (5.3%); reoperation 2/38 (5.3%); transfusion 2/38 (5.3%)	None reported (0/8 transplanted; no DGF or graft loss)	None (0/38)	NR (outcomes reported to 24 months)
Hajjar et al., 2021 [17]	NR (SSWL in 76.3%; 31/80 transplanted)	31/80 (39%); median 16.7 months after LSG	8 refused listing post-LSG: cardiovascular comorbidity (4), compliance (2), advanced cancer (1), unspecified (1)	9/80 (11.3%): pulmonary oedema (1), stroke (1), haemorrhage (3), dehydration (3), AF (1), urinary retention (1)	NR	7/80 died post-LSG before KT; 1 death >5 y post-KT	30.3 months (median, post-LSG)
Yemini et al., 2019 [18]	21/24 reached pathway (16 transplanted + 5 waitlisted)	16/24 (67%); mean 1.5 years after surgery	Of 8 not transplanted: 5 waitlisted, 2 died, 1 removed from list (new malignancy)	No intra-operative complications; 1 staple-line leak (fatal-see mortality)	2/16 delayed graft function (ATN); 1 acute rejection (steroid-treated)	2/24 (8.3%): staple-line leak/mediastinitis (day 21, surgery-related); sepsis unrelated (18 mo)	47 months (mean)
Thomas et al., 2018 [19]	25/31 (80.6%) waitlisted; median 11 months after surgery	14/31 (45%); median 33 months after surgery	Of those not transplanted: 3 in workup, 3 waitlisted, 5 transferred, 3 delisted (medical; 1 weight regain), 2 not pursuing, 1 never eligible	8/31 (25.8%), mostly minor: nausea/vomiting/pain (5; 1 laparotomy), swelling (1), stricture (1), hyperperistalsis-RYGB reversal (1)	5/14 (35.7%): ruptured graft, ureteral leak + ruptured renal artery, wound infection, perinephric haematoma, lymphocele	0% peri-operative; 0/14 post-transplant deaths	NR (post-transplant follow-up median 36 months)

Abbreviations: AF, atrial fibrillation; ATN, acute tubular necrosis; BS, bariatric surgery; DD, deceased donor; DGF, delayed graft function; eGFR, estimated glomerular filtration rate; ESRD, end-stage renal disease; GI, gastrointestinal; KT, kidney transplant; LD, living donor; LSG, laparoscopic sleeve gastrectomy; MBS, metabolic and bariatric surgery; NR, not reported; RYGB, Roux-en-Y gastric bypass; SG, sleeve gastrectomy; SSWL, successful surgical weight loss.

## Data Availability

All data generated or analysed during this study are included in this article. Further enquiries can be directed to the corresponding author.

## Supplementary Materials

The following supporting information can be downloaded at: https://www.mdpi.com/article/10.3390/jcm15145500/s1; Table S1. Per-timepoint anthropometric and renal data for the MBS group. Figure S1: Focused Flowchart of Studies identified for focused review [31].

## Abbreviations

The following abbreviations are used in this manuscript:

BMI	Body mass index
MBS	Metabolic bariatric surgery
LSG	Laparoscopic sleeve gastrectomy
OAGB	One- anastomosis gastric bypass
IQR	Inter-quartile range
ESRD	End- Stage Renal Disease
CKD	Chronic Kidney Disease
RT	Renal Transplantation
TWL	Total Weight Loss
GLP-1	Glucagon-like Peptide-1
EPS	Encapsulating Peritoneal Sclerosis
