# Supplementary material for: Metabolic Bariatric Surgery to Facilitate Access to Renal Transplantation for Patients with Obesity and End- Stage Renal Disease: A National Case Series and Focused Review
Source: J Clin Med. 2026 Jul 14;15(14):5500. doi: 10.3390/jcm15145500 (PMC13413131; doi:10.3390/jcm15145500)
Supplement: Supplementary file 1 [file jcm-15-05500-s001.zip › Supplementary Files.pdf]

Supplementary Files

Table S1. Per-timepoint anthropometric and renal data for the MBS group.

	**Patient 1**	**Patient 2**	**Patient 3**	**Patient 4**	**Patient 5**
Starting BMI (kg/m²)	51.1	49	59.8	43.3	43.7
Weight at MBS (kg)	110	118	149.7	120.6	107.7
BMI at MBS (kg/m²)	42.9	43	43.8	40.3	40
Weight at listing (kg)	74	91	–	93	85
BMI at listing (kg/m²)	28.9	33.5	–	31.1	31.6
Weight at transplant (kg)	68	–	–	87.2	83
BMI at transplant (kg/m²)	26.6	–	–	29.1	29.7
Creatinine at last follow up (μmol/L)	68	548	1100	86	156
eGFR at last follow up (mL/min/1.73 m²)	>60	9	4	56	35

MBS: metabolic bariatric surgery, BMI: body mass index, eGFR: estimated glomerular filtration rate. – indicates timepoint not reached (patient not listed or not transplanted).

Figure S1: Focused Flowchart of Studies identified for focused review

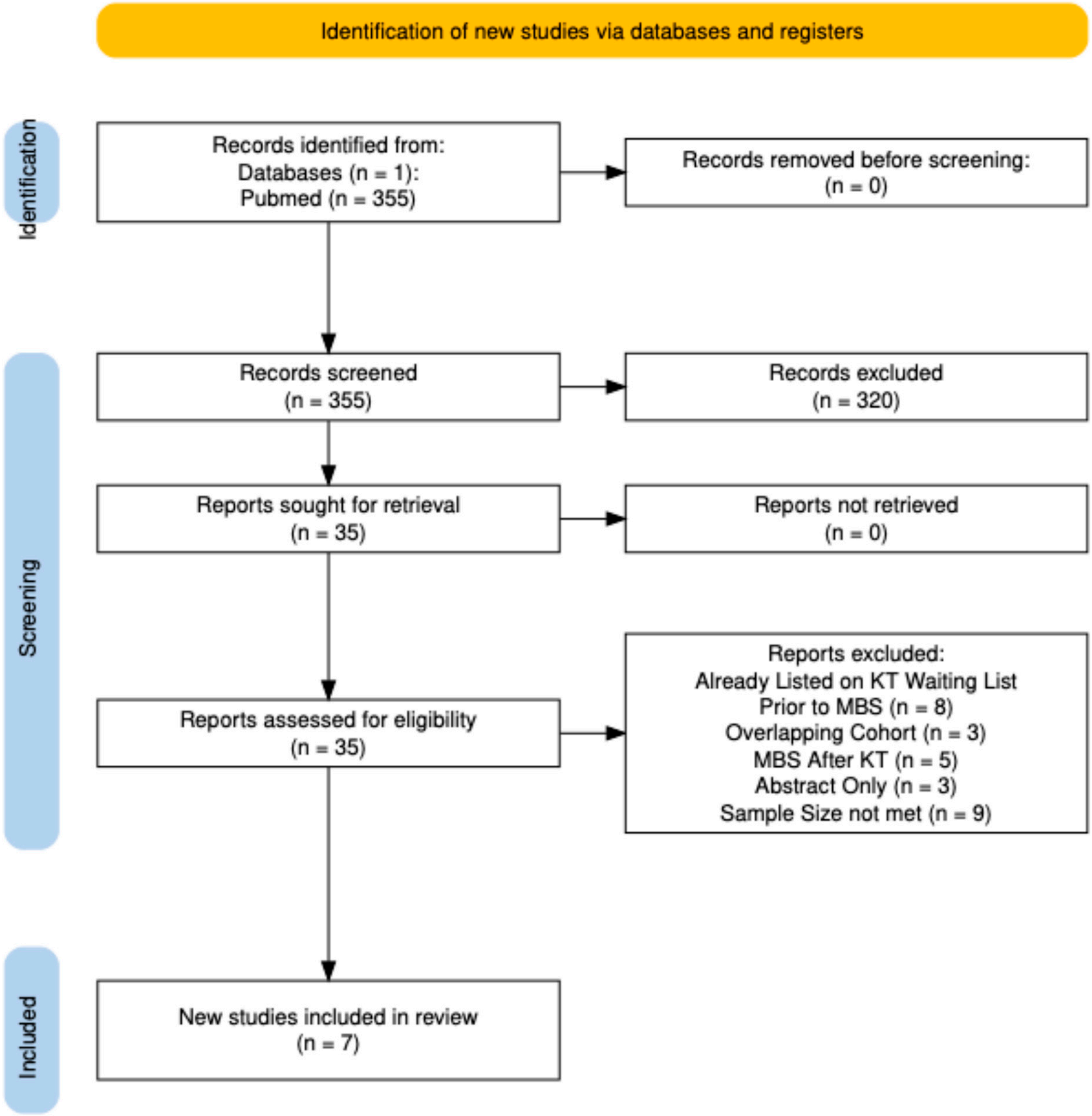

Haddaway, N. R., Page, M. J., Pritchard, C. C., & McGuinness, L. A. (2022). PRISMA2020: An R package and Shiny app for producing PRISMA 2020-compliant flow diagrams, with interactivity for optimised digital transparency and Open Synthesis Campbell Systematic Reviews, 18, e1230. https://doi.org/10.1002/cl2.1230
